# List of Physicians Registered in the Clerk’s Office of the County of Erie

**Published:** 1880-11

**Authors:** 


### LIST OF PHYSICIANS REGISTERED IN THE CLERK'S OFFICE OF THE COUNTY OF ERIE

We have taken great pains to prepare a complete list of those who have complied with the New Medical Law. Comment is unnecessary at this time. We shall take occasion to refer to this subject in a future number of the Journal.

| Name. | Graduate of | Date. |
|---|---|---|
| Abbey, Emery C. | Eclectic Med. College, Phila. Pa. | April, 1863. |
| Abbott, Frank A. | Med. Dept. University of Buffalo | Feb. 1866. |
| Armstrong, J. Stone | Jefferson Med. College, Phila. | Mar. 12, 187–. |
| Abbott, George | Buffalo Med. University | Feb. 25, 1852. |
| Allen, Jabez | Castleton Med. College, Vt. | Nov. 20, 1833. |
| Appleby, H. T. | Erie County Homœopathic Med. Society | |
| Allen, Daniel W. | Med. Dept. University of Buffalo | Feb., 1876. |
| Brown, George L. | Harvard Med. College, Mass | June 20, 1870. |
| Brecht, F. E. L. | University of Buffalo | Feb. 22, 1872. |
| Briggs, Albert H. | Med. Department University of Buffalo | Feb. 20, 1871. |
| Banta, Rollin L. | Med. Department University of Buffalo | Feb. 20, 1871. |
| Brayton, Samuel N. | Coll. of Phys. & Surg. New York City | Mar., 1861. |
| Beals, Herbert J. | N. Y. Homœopathic Med. College | Mar., 1878. |
| Barker, Arthur M | Med. Department University of Buffalo | Feb. 20, 1877. |
| Boardman, John | University of Pennsylvania | Apr. 2, 1853. |
| Bartow, Bernard | Med. Department University of Bnffalo | Feb. 25, 1874. |
| Bailey, Daniel A. | Med. Department University of Buffalo | Feb., 1872. |
| Barnes, Edwin R. | Long Island Coll. Hosp., Brooklyn, N. Y. | July, 1865. |
| Brooks, Abel D. | Eclectic Med. Coll. of the City of New York, | May 28, 1872. |
| Brooks, A. J. | Albany Medical College | 1860. |
| Baethig, Henry | Hahneman Med. College, of Philadelphia | Mar. 9, 1870. |
| Barrett, Willian C. | University of Buffalo | Feb., 1880. |
| Bissell, Elias L. | University of Michigan | Mar. 4, 1861. |
| Bidaman, Weston D. | University of Buffalo | Feb. 26, 1878. |
| Bull, Louis A. | University of Buffalo | Feb. 25, 1879. |
| Bull, A. T. | N. Y. University, Medical Department | Mar., 1848. |
| Berkes, Mary | University of Buffalo | Feb. 22, 1880. |
| Burt, Franklin | Coll. of Phys. and Surg. of Ontario, Canada | May 21, 1878. |
| | And University of Toronto | June 10, 1879. |
| Bartlett, Frederick W. | New York Medical College | Mar., 1854. |
| Bonnar, John D. | University of Trinity College, Toronto | May, 1878. |
| Bourne, C. W. | Castleton Medical College, Vt. | June 12, 1860. |
| Boies, Loren F. | Med. Dept. University of Buffalo | Feb. 25, 1868. |
| Broardt, Peter | Homeopathic Med. College of Erie Co. | Dec. 6, 1876. |
| Burwell, Geo. N. | University of Pa., in Philadelphia | April, 1843. |
| Blakely, Isaac C. | Licensed by University of Buffalo | |
| Colton, Henry E. | Cleveland Homeopathic College | Feb. 14, 1872. |
| Crane, Lucien D. | Licensed by Eclectic Med. Coll. of Genesee Valley District | June 4, 1879. |
| Crittenden, S. C. | Medical College, Pa. | 1857. |
| Clothier, Wm. P. | Med. Dept. University of Buffalo | Feb. 23, 1875. |
| Curtiss, Alexander M. | Pulte Med. College, Cincinnati | May 24, 1877. |
| | Homeopathic Medical College | Feb. 28, 1878. |
| Cronyn, John L. C. | Med. Dept. University of Buffalo | Feb., 1876. |
| Cronyn, John | University of Toronto | April, 1850. |
| | State Censors of N. Y. | June, 1860. |

| Name. | Graduate of | Date. |
|---|---|---|
| Crane, Ory Philo | Eclectic Medical Society of the Genesee Valley District | June, 1879. |
| Cary, Charles | Med. Dept. University of Buffalo | Feb., 1875. |
| Colyer, Charles W | Med. Dept. University of Buffalo | Feb., 1862. |
| Champlin, Chas. G | Med. Dept. University of Buffalo | Feb. 24, 1880. |
| Campbell, Robert E | University of Michigan | Mar. 25, 1866. |
| Clark, C. G | University of Michigan | Mar. 13, 1864. |
| Callahan, Thos. H | Eclectic Med. Coll. of Philadelphia, Pa | Jan. 21, 1863. |
| Daniels, Clayton M | University of Buffalo | Feb. 25, 1880. |
| Dagenais, Alphonse | Victoria University, Canada | May 10, 1867. |
| Diehl, Conrad | University of Buffalo | Feb., 1866. |
| Davidson, A. R | Med. Dept. University of Buffalo | Feb., 1878. |
| Dorland, Elias T | University of Michigan | Mar. 24, 1854. |
| Dye, John H | Eclectic College of N. Y. City | Feb. 29, 1873. |
| Dayton, Charles L | Buffalo Medical University | Oct., 1853. |
| Devening, Daniel | University City of N. Y. Med. Dept | Mar. 6, 1846. |
| Daggett, Byron H | University of Buffalo | Feb., 1867. |
| Danforth, W. A | Rush Med. College, Chicago, Ill | Feb. 3, 1869. |
| Dayton, L. P | Geneva Med. College | Feb., 1846. |
| Dorr, Samuel G | Buffalo Med. University | Feb., 1875. |
| Denny, Mrs. Louisa | Victoria College, Kingston, Ont | Oct. 10, 1845. |
| Dambach, John | Buffalo Med. College | Feb , 1868. |
| Erb, Peter | University of Michigan | Mar. 26, 1879. |
| Ellwood, Henry S | Med Dept University of Buffalo. | Feb , 1868. |
| Elliott, Justin C | Med. Dept. University of Buffalo | Feb., 1851. |
| Earl, W. C | Bellevue College, N. Y | Mar. 3, 1864. |
| Eisbein, David | University of Buffalo | Feb. 26, 1878. |
| Farrington, Edwin H | Buffalo Med. College | Feb. 27, 1867. |
| | Bellevue Med. College | Feb. 28, 1875. |
| Fowler, Joseph | Med. Dept. University of Buffalo | Feb. 25, 1873. |
| Foster, Hubbard A | Med. Dept. Harvard Univ. Boston. Mass | Mar. 8, 1877. |
| Frost, Henry C | Cleveland Homeopathic Med. College | Feb. 11, 1874. |
| Farrington, D. H | Buffalo Med. College | Feb., 1863. |
| Folwell, M. B | Med. Dept. University of Buffalo | Feb., 1867. |
| Gould, William | Med. Dept. University of Buffalo | Feb. 27, 1850. |
| Granger, William D | Bellevue Hospital Medical College | Mar , 1879. |
| Gay, Charles C. F | Berkshire Medical Institution | 1846. |
| Gail, Wm. H | Medical Department University of Buffalo | Feb., 1864. |
| Greene, Joseph C | Albany Medical College | June 12, 1855. |
| Greene, S. S | Medical College of the University of N. Y | Mar., 1864. |
| Gueso, Carl H | Buffalo Medical University | Feb. 25, 1880. |
| Grove, Benjamin H | Medical University of Buffalo | Feb , 1880. |
| Greene, Walter D | University of Buffalo | Feb. 26, 1878. |
| Gigolsky, Bernhard | Königsberg Hospital, Prussia | Nov. 13, 1871. |
| Gipple, F. M | University of Buffalo | Feb. 25, 1880. |
| Hancock, Alexander S. | Toronto School of Medicine | Apr. 25, 1868. |
| | College of Phys. and Surg., Ontario, Canada. | Aug. 30, 1878. |
| Hubbell, Alvin A | University of Buffalo | Feb. 23, 1876. |
| Hoffmeyer, John A | Medical Department University of Buffalo | Feb. 25, 1880. |
| Haberstro, Joseph | Medical Department University of Buffalo | Feb , 1880. |
| Hoyer, Frederick F | Medical Department University of Buffalo | April, 1849. |
| Hauenstein, Jno | Geneva Medical College, State of N. Y | Jan., 1844. |
| Howard, Charles F | Medical Department University of Buffalo | Feb., 1878. |
| Harrington, D. W | Medical Department University of Buffalo | Feb., 1871. |
| Harvey, C. W | Medical Department University of Buffalo | 1838. |
| Harvey, Leon F | Jefferson Medical College, Pa | Mar., 1859. |
| Hill, John D | Medical Department University of Buffalo | Apr., 1849. |
| Hartwig, Marcell | Fred. Wilhelm's University at Berlin, Prussia | 1873. |
| | State Censors of the State of New York | 1879. |

| NAME. | GRADUATE OF | DATE. |
|---|---|---|
| Howe, Lucien | Royal College of Surgeons, England | July 23, 1873. |
| | Bellevue Medical College, Long Island | 1872. |
| Hurd, S. Wright | Hahnemann Med. College of Philadelphia | Mar. 10, 1880. |
| Hoxsie, A. C | Cleveland Homeopathic Medical College | Feb., 1864. |
| Hopkins, H. R. | Medical Department University of Buffalo | Feb., 1867. |
| Hebenstreit, Robert | Medical Department University of Buffalo | Feb., 1872. |
| Halbert, John S | Buffalo Medical College | Feb., 1872. |
| Hill, Clayton L | Buffalo Medical College | Feb., 1864. |
| Heinemann, J. D | Cleveland Homeopathic Hosp. College | Feb. 17, 1875. |
| Henderson, Sallie B | College of Phys. and Surg, Buffalo, N. Y | Feb. 26, 1880. |
| Hoyt, Horace | Buffalo Medical College | June, 48. |
| Johnson, Thomas M | Buffalo Medical College | Feb., 1861. |
| Jackson, W. H | South Carolina University | June 4, 1873. |
| Johnston, E. E. | Buffalo Medical College | Feb. 26, 1880. |
| Krug, Julius F | Medical College of Indiana, Department of Butler University | Mar. 1, 1878. |
| Kilbourne, H. S | University of Michigan | Mar., 1865. |
| Kenyon, William B | Homeopathic College of Philadelphia | Mar., 1874. |
| King, J. E | University of Buffalo | June, 1847. |
| Krombein, Louis E | Buffalo Medical College | Feb., 1860. |
| Kenyon, Lorenzo M | Homoepathic Medical College of Missouri | Feb, 1875. |
| Keene, Jos. W | Med. School of Harvard University | June 27, 1878. |
| Kamerling, Andrew | Med. Dept. University of Buffalo, N. Y | Feb., 1864. |
| Lanigan, John A | University of the City of New York | Feb., 1876. |
| Little, Edward | Med. Dept. University of Buffalo | Feb., 1862. |
| Long, William E | Cleveland Homeopathic Hospital College | Feb. 25, 1880. |
| Lothrop, Thomas | University of Michigan | Mar. 25, 1858. |
| Loomis, Horatio N | The Faculty of the Medical College at Fairfield, Herk. Co., N. Y | 1828. |
| Lynde, U. C | Buffalo University | Feb., 1859. |
| | And Jefferson Med. Coll | Feb., 1866. |
| Lothrop, Benj. L | Bellevue Hospital Med. College | Feb., 1872. |
| Lewis, Geo. W | Med. Dept. University of New York | Mar., 1850. |
| Lewis, F. Park | Puelte Med. College | 1876. |
| Lou See On | Lou Com Chong, a duly designated authority in Canton, China, July 28, in the 34th year of the reign of Ton Kong, Emperor of China | |
| McGill, W. D | H. H. College, Cleveland, O | Feb. 25, 1880. |
| McNeil, Dugald | Med. Dept. University of Buffalo | Feb. 20, 1871. |
| Myers, Reuben S. | Med. Dept. University of Vermont | July 1, 1875. |
| Mynter, Herman | Med. Society of the State of N. Y | June 1, 1875. |
| McPherson, Geo. W | University of Michigan | Mar. 28, 1866. |
| McMichael, L. D | Eclectic Med. College of Philadelphia, Pa | Jan. 23, 1868. |
| Meisburger, Wm | University of Freiburg, Germany | |
| Miller, John G. | University of Buffalo | Feb., 1876. |
| Moody, Mary B | University of Buffalo | Feb., 1876. |
| Mixer, S. F | Med. Dept. Yale Coll. and Phys. and Surg. N. Y. City | Mar., 1847. |
| McDonald, Samuel | Buffalo Med. College | Feb. 23, 1859. |
| Miner, Julius F | Albany Med. College | Jan. 26, 1847. |
| Mackay, Gustavus E | Med. Dept. University of Buffalo | Feb. 23, 1866. |
| McCrea, Philip A | Med. Dept. University of Buffalo | 1875. |
| Marcley, James J | Coll. of Phys. and Surg., N. Y. City | Feb., 1873. |
| Matzinger, Charles | University of Wein, Germany | Jan. 17, 1830. |
| Murray, William D | National Med. College. | Mar., 1859. |
| Murray, Henry B. | Bellevue Hospital Medical College | Feb., 1868. |
| Moore, Samuel H | College of Phys. and Surg., New York | Mar. 14, 1867. |
| Nichell, Henry | Medical Department University of Buffalo | Feb. 25, |

| Name. | Graduate of | Date. |
|---|---|---|
| Nott, S. E. S. H | Castleton Medical College | Nov., 1844. |
| O'Connell, Patrick M. | Missouri Medical College | Mar. 2, 1877. |
| Osborne, Nehemiah | Buffalo University | Feb., 1863. |
| O'Brien, Edward C.W. | Buffalo Medical College | Feb., 1867. |
| Osborne, F. G. | Buffalo Medical College | Feb.,'68 or '69. |
| Pynchon, Edwin | Medical College of Ohio, Cincinnati, O. | Mar. 2, 1876. |
| Pohle, John D. A. | Cleveland Medical College | Feb. 2, 1880. |
| Peterson, Frederick | Med. Department University of Buffalo | Feb., 1880. |
| Potter, Samuel | Academy of Medicine, Vt. | Nov. 12, 1836. |
| Pierce, Franklin D. | Univ. of the City of N. Y., Med. Dep't, | Feb. 19, 1878. |
| Pattison, Geo. W. | University of Buffalo | Feb. 24, 1869. |
| Packwood, W. J. | Buffalo Medical College | Feb., 1876. |
| Pennell, Ezra | Buffalo Medical College | Feb. 27, 1867. |
| Parker, L. P. L. | Buffalo Medlcal College | Feb. 22, 1854. |
| Phelps, Wm. C. | Med. Department University of Buffalo | Feb. 22, 1866. |
| Pierce, Ray V. | Eclectic Med. College of Cincinnati, O. | Feb. 15, 1865. |
| Petsch, Charles F. | Cleveland Homœopathic College | Feb., 1870. |
| Pettit, John A. | Buffalo Medical College | Feb. 24, 1874. |
| Parnenter, W. L. | Homœopathic College of Cleveland, O. | Feb. 12, 1873. |
| Rowe, George G. | University of Toronto | June 10, 1879. |
| Ring, Charles A. | Med. Department University of Buffalo | Feb 26, 1878. |
| Rochester, Thomas F. | Med. Dep't University of Pennsylvania | Feb., 1848. |
| Richmond, Ira | New York Medical College | June, 1856. |
| Rogers, Henry W. | Buffalo Medical College | Feb. 25, 1880. |
| Retel, Casper | License by College of Physicians and Surgeons, Buffalo, 25 years' practice and candidated for graduation | Oct. 2, 1880. |
| Ring, William | University of Buffalo | June 16, 1847. |
| Rouse, M. D. | Med. Dep't University of Pennsylvania | Mar., 1866. |
| Stumpf, Daniel B. | Cleveland Homœopathic College | Feb., 1876. |
| Schade, Louis C. F. | Licensed by the Medical Society of the State of New York | July 22, 1876. |
| Shaw, Merrill H. | Medical College at Geneva, N. Y. | 1839. |
| Stanley, Fred. G. | N. Y. Medical College, New York City | Mar., 1861. |
| Stockton, Chas. G. | Med. Department University of Buffalo | Feb., 1878. |
| Sisson, Perry | American Health College, Cincinnati, O. | Oct., 1876. |
| Storck, Edward | University of Michigan | Mar., 1854. |
| Searles, Macy B. | Buffalo Medical College | Feb. 23, 1869. |
| Simson, J. R. | Hom. Hosp. College, Cleveland, O. | Feb., 1880. |
| Stearns, George R. | N. Y. Homœpathic Medical College | Feb. 28, 1878. |
| Smith, Lee Herbert | University of Buffalo | Feb., 1877. |
| Slacer, William H. | Medical University of Buffalo | Feb. 22, 1873. |
| Storck, Eugene E. | Medical University of Buffalo | Feb., 1878. |
| Strong, Phineas H. | Albany Medical College | Mar., 1839. |
| Samo, J. B. | College of Phys. aad Surg., New York City | Mar. 28, 1832. |
| Sonnick, P. L. | University of Wurzburg, Germany | Apr., 1853. |
| Smith, James S. | University of Buffalo | Feb. 25, 1862. |
| Seymour, Abby Janet | Hahneman College, Chicago, Ill. | Mar., 1873. |
| Sloan, James | Medical Department University of Buffalo | Feb. 22, 1870. |
| Sweetland, Fred W. | Medical Department University of Buffalo | Feb., 1878. |
| Strong, Orville C. | Medical Department University of Buffalo | 1871. |
| Sperry, M. M. | Licensed by S. W. Wetmore. | 1880. |
| Shaw, M. B. | Buffalo Medical College | 1866. |
| Stevenson, James D. G. | 44 years' practice, of which over 30 years has been in Buffalo, Erie Co., N. Y. | |
| Tyler, Clarence A. | Western Reserve College, Hudson, O. | Mar. 12, 1874. |
| Turner, A. F. | Cleveland Homeopathic Hosp. Coll., O. | Mar., 1875. |
| Thoma, L. Otto | Med. Department University of Buffalo, | Feb., 1870. |
| Tobie, E. | Geneva Medical College, N. Y. | Feb. 10, 1855. |

| Name. | Graduate of | Date. |
|---|---|---|
| Treffehn, H.. | University of Halle, Prussia | 1846. |
| Thompson, Justin G. | University of Michigan | Mar. 27, 1861. |
| Turver, W. W.. | Bellevue Hospital Med. College, N. Y. | Mar., 1879. |
| Thomas Cesar.. | Grossherzoglich Badische, Albert Ludwig's Hochschule | Dec., 1877. |
| Taylor, T. B. | New York Central Med. Association | Sept., 1878. |
| Volker, Louis C | Med. Dept. University of Buffalo | Feb , 1877. |
| Van Peyma, W. | University of Toronto | April, 1850. |
| | State Curators of N. Y | June, 1860. |
| Van Peyma, J. W.. | University of Goningen, Holland | 1841. |
| Von Schulenberg, E . | Coll. of Physicians & Surg., Buffalo, N.Y. | Feb. 20, 1880. |
| Vaughn, Frank O.. | Buffalo Med. Univ. State of New York | Feb. 25, 1880. |
| Van Peyma, P. W. | Med. Dept. University of Buffalo | Feb., 1872. |
| Walsh, John J. | Med. Dept. University of Buffalo | Feb. 25, 1871. |
| Weet, Elroy Sabin. | Med. Dept. University of Buffalo | Feb. 24, 1874. |
| Wyckoff, Cornelius C.. | Med. Dept. University of Buffalo | June, 1848. |
| Wiggins, D. B.. | P. M. College of Cincinnati, O. | Feb. 16, 1846. |
| Wetzel, Charles H. | Med. Dept. University of Buffalo | Feb., 1876. |
| Walton, John Tompkins, | University of Pennsylvania at Phila., Pa. | Mar. 29, 1856. |
| Willoughby, Matthew.. | Med. Dept. University of Buffalo | Feb., 1867. |
| White, Russel J. | New York Eclectic Medical College | Feb. 4, 1875. |
| Wenz, Julius. | University of Buffalo | Feb. 21, 1865. |
| Wetmore, Samuel W.. | Med. Dept. University of Buffalo | Feb., 1862. |
| Warren, Samuel H. | Buffalo Medical College | Feb. 25, 1880. |
| Wall, Charles A. | University of Buffalo | Feb., 1879. |
| White, James P. | Jefferson Medical College of Phila. | Mar. 1834. |
| Wage, John F.. | Detroit Homeopathic College | Feb. 12, 1874. |
| Willet, Phebe | Col. of Phys. and Surg., Syracuse, N. Y. | June 28, 1876. |
| Wolsey, C. F. | Ten years' Practice and Candidate for Graduation Col. Phys. & Surg., Buffalo, | |
| Wende, Ernest. | Med. Dept. University of Buffalo | Feb. 28, 1876. |
| Waldruff, E. C.. | Buffalo Medical University | Feb , 1880. |
| Whitten, Clarke | American Medical College of St. Louis | Jan. 30, 1874. |
| Zielinski, Jacob | Possen Medical Institute, Possen, Prussia | 1864. |

